# Identifying New Factors Associated With Cognitive Decline and Delirium After Transcatheter Aortic Valve Implantation: A Study Protocol

**DOI:** 10.3389/fcvm.2021.657057

**Published:** 2021-08-11

**Authors:** Erica S. Ghezzi, Peter J. Psaltis, Tobias Loetscher, Daniel Davis, Joseph Montarello, Jerrett K. Lau, Sinny Delacroix, Alice Bourke, James McLoughlin, Megan Keage, Hannah A. D. Keage

**Affiliations:** ^1^Cognitive Ageing and Impairment Neurosciences Laboratory, Justice and Society, University of South Australia, Adelaide, SA, Australia; ^2^Vascular Research Centre, Lifelong Health Theme, South Australian Health and Medical Research Institute, Adelaide, SA, Australia; ^3^Adelaide University Medical School, University of Adelaide, Adelaide, SA, Australia; ^4^Department of Cardiology, Royal Adelaide Hospital, Central Adelaide Local Health Network, Adelaide, SA, Australia; ^5^MRC Unit for Lifelong Health and Ageing Unit at UCL, London, United Kingdom; ^6^Department of Geriatric and Rehabilitation Medicine, Royal Adelaide Hospital, Central Adelaide Local Health Network, Adelaide, SA, Australia; ^7^College of Nursing and Health Sciences, Flinders University, Adelaide, SA, Australia; ^8^Centre for Neuroscience of Speech, The University of Melbourne, Melbourne, VIC, Australia; ^9^Department of Audiology and Speech Pathology, The University of Melbourne, Melbourne, VIC, Australia

**Keywords:** TAVI, delirium, cognitive decline, risk prediction, transcatheter aortic valve implantation, post-operative cognitive decline

## Abstract

**Background:** Transcatheter aortic valve implantation (TAVI) has become the standard-of-care for treatment of severe symptomatic aortic stenosis and is also being increasingly recommended for low-risk patients. While TAVI boasts positive post-procedural outcomes, it is also associated with cognitive complications, namely delirium and cognitive decline. There is a pressing need for accurate risk tools which can identify TAVI patients at risk of delirium and cognitive decline, as risk scores designed for general cardiovascular surgery fall short. The present effect-finding exploratory study will assess the utility of various measures in the context of aging and frailty in predicting who will and who will not develop delirium or cognitive impairment following TAVI. The measures we propose include gait, visual symptoms, voice, swallowing, mood and sleep.

**Methods:** This is an observational prospective cohort study focused on identifying pre-procedural risk factors for the development of delirium and cognitive decline following TAVI. Potential risk factors will be measured prior to TAVI. Primary outcomes will be post-procedure cognitive decline and delirium. Secondary outcomes include activities of daily living, quality of life, and mortality. Delirium presence will be measured on each of the first 2 days following TAVI. All other outcomes will be assessed at 3-, 6-, and 12-months post-operatively. A series of logistic regressions will be run to investigate the relationship between potential predictors and outcomes (presence vs. absence of either delirium or cognitive decline).

**Discussion:** This study will assess the strengths of associations between a range of measures drawn from frailty and aging literature in terms of association with cognitive decline and delirium following TAVI. Identified measures can be used in future development of TAVI risk prediction models, which are essential for the accurate identification of cognitive at-risk patients and successful application of pre-procedural interventions.

**Clinical Trial Registration:** This trial is registered with the Australian New Zealand Clinical Trials Registry. [https://bit.ly/2PAotP5], [ACTRN12618001114235].

## Introduction

Severe symptomatic aortic stenosis is a heart condition which affects one-in-ten people over the age of 75 (1). Surgical aortic valve replacement was traditionally the usual treatment for this condition, yet approximately one third of patients are unsuitable for surgery due to older age or comorbidities (2). In 2002, transcatheter aortic valve implantation (TAVI) was introduced as a non-surgical alternative for the treatment of severe symptomatic aortic stenosis and has since become the standard of care in the large number of patients with prohibitive surgical risk (3). Recommendation for TAVI treatment has been extended to low-risk patients in recent years (4, 5). During a TAVI procedure, a new valve is positioned into the heart from a blood vessel in the groin or an anterograde transapical approach, thus circumnavigating the need for cardiopulmonary bypass and sternotomy (6).

While TAVI is associated with positive post-procedural outcomes, such as lower mortality and improvements in quality of life (7–9), cognitive complications including delirium and cognitive decline are common (10, 11). Delirium is an acute and fluctuating disorder of deficits in attention and arousal (12). A previous meta-analysis by our group found that one-in-four TAVI patients experience delirium in the days following their procedure (10). In older individuals delirium is associated with a range of poor outcomes including functional decline, loss of independence, cognitive decline and dementia, institutionalization and death (13, 14). Furthermore, another meta-analysis by our group demonstrated that cognitive decline is seen in up to 14% of patients in the year following TAVI (11). Cognitive decline can lead to clinically significant cognitive impairments including mild cognitive impairment (MCI) and dementia (15, 16).

Given the frequency of these poor cognitive outcomes, there is a pressing need for tools and methods which accurately identify those at increased risk of developing delirium and cognitive decline following TAVI. Identification of those at risk allows for the implementation of pre-procedural education, multicomponent intervention and appropriate community follow-up in these patients (17). The current study is an effect-finding study, which looks to examine the utility of several potential factors related to incident delirium and cognitive decline. These include those that were identified by our meta-analysis (10), are currently used in delirium risk tools for general cardiac patients (18, 19), and novel factors previously unexplored in this context, identified from the broader aging and frailty literature. Notably, previous tools (18, 19) have focused on delirium not cognitive decline. These factors related to delirium within previous tools and our meta-analysis are outlined in Table 1.

**Table 1:** Risk factors from existing prediction models for post-cardiac procedure delirium and from meta-analysis of post-TAVI delirium predictors.

**Variable**	**Katznelson et al. (18)** ^*^	**Rudolph et al. (19)** ^*^	**Tilley et al. (10)** ^***†***^
**Pre-procedural variables**			
Abnormal albumin		✓	
Age	✓		
Atrial fibrillation			✓
Cognitive impairment		✓	✓
Coronary artery disease			✓
Depression	✓	✓	
EuroSCORE			
Hypertension			✓
Peripheral vascular disease			✓
Renal dysfunction	✓		✓
Statin use (protective)	✓		
Stroke history		✓	✓
**Intra-procedural variables**			
Valve access type			✓

* *Prediction model for cardiac procedures*.

† *Meta-analysis for TAVI*.

To our knowledge, there are no risk prediction models for post-procedural cognitive decline in TAVI patients or cardiac patients generally. A recent meta-analysis (11) reported that the use of cerebral embolic protection devices and pre-procedure cognitive impairment reduced the risk of post-TAVI cognitive decline. Within the context of an aging population, there is a general rising demand for pre-operative risk stratification of older adults to aid informed consent and the guiding of patient expectations (20). Risk scores such as European System for Cardiac Operative Risk Evaluation (EuroSCORE) and Society of Thoracic Surgery (STS) are currently widely used as part of pre-TAVI evaluation. However, these scores were developed for conventional cardiovascular surgery and are limited in their predictive utility for TAVI patient populations (21–25).

In the absence of specific risk tools for TAVI, measures of frailty have been recommended (26–28) and have demonstrated reasonable success in predicting adverse post-operative outcomes (29, 30). Frailty is a problematic expression of aging which represents increased vulnerability to sudden health changes in response to only minor stressor events (31). This clinical condition, affecting between 25 and 50% of those over 85 years old, develops as a result of accelerated age-related decline across multiple physiological systems (31). A growing body of evidence over recent years has demonstrated that frailty and other functional measures are predictive of worse outcomes, such as mortality, after TAVI (28, 32–39). Specific to delirium, a meta-analysis has demonstrated a significant association between frailty and delirium, with frail individuals carrying a 2-fold increased risk of subsequent delirium (40). In TAVI patient populations, there have been conflicting accounts on the relationship between frailty and post-operative delirium (41–43). This lack of consensus may be due to differences in the frailty measures employed, and lack of clarity regarding which features of frailty carry the most predictive utility in a TAVI sample. Research has demonstrated that frailty increases risk of incident Alzheimer's disease and cognitive decline (44). Only one study (45) has investigated differences in pre-procedural frailty between those who did and did not develop cognitive decline following TAVI, with no difference found. However, once again, only one frailty measure was used, and individual components of frailty were not investigated.

The concept of frailty emerged from epidemiological studies and measures have generally been composed of relatively blunt items (e.g., the presence or absence of a health issue) (31). At a population-based level, these frailty measures have shown to predict mortality and morbidity well (39). In smaller clinical samples, there is great opportunity to develop more sensitive measures that capture frailty and aging processes. We propose to assess the utility of various measures in the context of aging and frailty in predicting who will and who will not develop delirium or cognitive decline post-TAVI. It is not our purpose to develop a new measure of frailty *per se*, but rather employ knowledge of aging and frailty processes to inform our selection of new risk measures of delirium and cognitive decline in TAVI patients. The measures we propose include gait, visual symptoms, voice, swallowing, mood and sleep. All of these have previously been associated with aging, dementia, cognitive decline, or neurodegeneration, and are easy to collect in clinical settings.

High dual-task gait cost (the change from single-task to dual-task gait speed) associates with a 2- to 3-fold increase in incident dementia risk in those with existing MCI (46). In TAVI populations, impaired mobility has also been related to post-operative delirium and reduced quality of life (33, 47). Research has shown eye blink rate to be significantly higher or lower in those with cognitive impairments. A higher blink rate has been found in MCI (48), while a lower blink rate has been reported in those with Parkinson's disease, as compared to controls (49). Visual symptoms could also have utility, particularly convergence, which can lead to diplopia (double vision) and is seen in neurodegeneration (50). Dysphagia (difficulty swallowing) has also been found in a wide variety of neurodegenerative disorders associated with aging, including Parkinson's disease (51). Finally, depression has been identified as a large predictor for delirium following cardiac procedures (52) and is included in existing prediction models (18, 19). We will include not only depression, but also more broad measures of neuropsychiatric symptoms including insomnia (53). In order to maximize potential clinical applicability, the novel symptoms we are introducing and investigating within the context of post-TAVI delirium and cognitive decline are easily implemented in clinical settings with minimal training and equipment.

In summary, this study will investigate associations between a wide range of factors and post-TAVI delirium and cognitive decline. Existing risk scores (STS and EuroSCORE), factors incorporated within current delirium risk prediction tools, and factors with reported associations to post-operative delirium will be evaluated. In addition to this, we will include a range of novel measures which are drawn from the literature of aging and frailty. The aim of this effect-finding study is to identify potential measures from which to develop risk prediction models for cognitive decline (at 3-, 6-, and 12-months post-procedure) and delirium specifically for TAVI patients. Such risk prediction models will aid in the identification of at-risk patients, allowing for earlier and targeted intervention to reduce the occurrence of delirium and cognitive decline following these procedures.

## Methods and Analysis

### Design

This study is an observational prospective cohort study focused on identifying pre-procedural risk factors for the development of delirium and cognitive decline following TAVI. Potential risk factors will be measured prior to TAVI (baseline). Cognitive function, activities of daily living, quality of life, and mortality outcomes will be assessed at 3-, 6-, and 12-months post-operatively. Delirium presence will be measured on each of the first 2 days following TAVI.

### Setting

Recruitment of participants occurs at one site: The Royal Adelaide Hospital. The Royal Adelaide Hospital is a public hospital located in the central business district of Adelaide, Australia. Delirium assessments (days one and two post-TAVI) are conducted where the participant is located on that day; in hospital or at their home (if they have been discharged). Baseline and follow-up testing sessions at 3-, 6-, and 12-months post-TAVI are conducted in participants' homes.

Recruitment commenced in May 2018 and is currently underway. The rate of recruitment is ~20 participants per year. However, the study was placed on hold for a significant portion of 2020 due to COVID-19 restrictions. To ensure fulfillment of the required sample size (*n* = 60, see Sample Size section), active recruitment is anticipated to continue until December 2021 (3.5 years in total), with study completion (end of 12-month follow-ups) in December 2022.

### Selection of Subjects

#### Inclusion Criteria

Participants are eligible if they are aged 60 years and over and have consented to undergo an elective TAVI procedure at the Royal Adelaide Hospital. They must live within a 1-h drive of the central business district of Adelaide. Normal hearing (with or without the use of aids) and proficient English language are also required. People with a clinical diagnosis of a neurodegenerative condition (including dementia) are also eligible for inclusion to reflect the general older population undergoing cardiovascular procedures. A different consent process, where a person responsible provides written and verbal consent, is undertaken for those with such diagnoses.

#### Exclusion Criteria

Participants will be ineligible for inclusion if they have: current or recent (within the past year) alcohol or substance abuse or dependence; recently (past month) used recreational drugs; a diagnosed learning disability; any conditions not typically associated with ischemic heart disease and likely to affect mobility, voice or swallowing to a significant degree (including congenital conditions, structural abnormalities, or cancer in pertinent regions of the body); already enrolled in a TAVI clinical trial; severe visual impairment.

### Sample Size

We want to capture any relationships between risk factors and cognitive outcomes which have potential clinical utility. As such, to detect a medium-large effect size or higher (*r* ≥ 0.4), with power = 0.8, α = 0.05, we computed [using G^*^Power statistical analysis software (54)] that a sample size of 44 is required. To account for attrition, a total of 60 participants will be recruited.

### Honoraria

Participants will receive a total of AUD$80 (4 × $20) worth of vouchers across the duration of the study to cover time and other costs associated with participation. One voucher is supplied to participants after the completion of the pre-TAVI baseline as well as after 3-, 6-, and 12-month post-TAVI follow-up sessions.

### Measures

#### Primary Outcomes

##### Cognitive Decline

Addenbrooke's Cognitive Examination III (ACE-III) will be used to identify cognitive decline. The ACE-III is a measure of global cognition, widely used as a dementia screening tool. It comprises five cognitive domains: attention, memory, verbal fluency, language, and visuospatial abilities. Possible scores range from 0 to 100, with higher scores representing better cognition. High sensitivity and specificity are found for the two suggested cut-offs, of 88 for MCI (sensitivity = 1.0, specificity = 0.96) and 82 for dementia (sensitivity = 0.92, specificity = 1.0) (55). The ACE-III also demonstrates good internal reliability (α = 0.88) and has three parallel versions which are all used within the study to allow for longitudinal cognitive testing (55). ACE-III version A will be used at baseline and 12-month follow-up, version B at 3-month follow-up, and version C at 6-month follow-up. The ACE-III score will be used to characterize participants as with or without cognitive decline (compared to baseline) at each follow-up timepoint (3-, 6-, and 12-months post-TAVI). Cognitive decline will be defined in the present study as a decline (from baseline to follow-up) of 4 or more points in ACE-III score. As an effect-finding study, this cut-off was chosen to weight toward false positives, and was based on one standard deviation (3.3) in ACE-III score previously reported for older adults (56).

##### Delirium

The presence of delirium will be assessed using the Confusion Assessment Method for the Intensive Care Unit (CAM-ICU) (57) for those in the ICU or the CAM (58) for participants in non-ICU wards or who have been discharged home. The CAM-ICU-7 (59) will be calculated to quantify delirium severity in the ICU. For participants in non-ICU wards or at home, the Memorial Delirium Assessment Scale (MDAS) (60) interview will be conducted to inform the scoring of the CAM and as a measure of delirium severity. The CAM and CAM-ICU comprise four main features of delirium: (1) acute onset and fluctuating course; (2) inattention; (3) disorganized thinking; and (4) altered level of consciousness. Altered level of consciousness will be assessed using the Observational Scale of Level of Arousal for the CAM, and the Richmond Agitation and Sedation Scale for the CAM-ICU. We define a positive diagnosis of delirium as the presence of features 1 and 2 and either 3 or 4 on the CAM or CAM-ICU. For any positive delirium diagnoses the delirium motor subtype (hypoactive, hyperactive, mixed, no subtype) will also be evaluated using a checklist by Meagher (61). Presence of delirium was considered as a positive diagnosis on either days 1 or 2 post-TAVI. All research team members conducting delirium assessments will be trained by qualified geriatricians (AB and DD).

#### Secondary Outcomes

##### Cognition (Continuous)

In addition to the dichotomous primary outcome of cognitive decline, change in cognition from baseline to each follow-up will be evaluated as a continuous secondary outcome. This change will be represented by *z*-score of the cognitive change score calculated for each follow-up timepoint in our sample (difference between an individual's score at follow-up and score at baseline). Change in ACE-III score, as well as change on tests conducted using Cambridge Neuropsychological Test Automated Battery (CANTAB) will be considered. A three-test battery will be run using the portable CANTAB testing system. The tests employed will be the Motor Orientation Task (MOT), Reaction Time (RTI), and Pattern Recognition Memory (PRM). The MOT will be used purely as tablet familiarization and, as such, no data will be analyzed from this task. Memory and attention are cognitive domains in which acute impairments are found following valve replacement (62). CANTAB allows for detection of changes in these more specific domains, rather than global cognition (ACE-III), with RTI primarily as a measure of attention and PRM primarily for visual memory. RTI and PRM provide multiple outcome measures. For both, a z-score for change (from baseline to follow-up) in each relevant outcome (e.g., reaction time, errors) will be calculated and then averaged, creating a composite z-score outcome for each test.

##### Additional Secondary Outcomes

Secondary outcomes also comprise quality of life and activities of daily living, which are measured using the EuroQol-5D (63) and Barthel Index of Activities of Daily Living (64), respectively, at 3-, 6-, and 12-months after TAVI. Mortality was also recorded as a secondary outcome at the same three timepoints post-procedure.

#### Potential Risk Factors

All potential factors to be measured are presented in Table 2, along with their duration and the time-points of the study at which they are assessed.

**Table 2:** Measures and assessments utilized throughout study period, with approximate collection time.

**Assessments**	**Duration (min)**	**Baseline**	**D1**	**D2**	**3 M**	**6 M**	**12 M**
**Cognitive Battery**							
Addenbrooke's Cognitive Examination-III (ACE-III) (56)^*^	15	X			X	X	X
Motor Screening Task (CANTAB)^*^	2	X			X	X	X
Reaction Time (CANTAB)^*^	3	X			X	X	X
Pattern Recognition Memory (CANTAB)^*^	5	X			X	X	X
**Delirium Daily Screen**							
Memorial Delirium Assessment Scale (MDAS)^*^ (60)	15		X	X			
Confusion Assessment Method Short Form (CAM) (58) or CAM-ICU (57) [and CAM-ICU-7 (59)]	3-5		X	X			
Motor Subtype Classification (61)	1		X	X			
**Activities of Daily Living**							
Barthel Index of Activities of Daily Living (64)	5	X			X	X	X
**Quality of Life**							
EuroQol-5D (63)		X			X	X	X
EuroQol-VAS (63)		X			X	X	X
**Mortality**							
**Electroencephalography**							
Resting Data (Note: related to a sub-study, not discussed further here)	30	X					
**Mood and Behavior**							
Geriatric Depression Scale (GDS) (65)	3	X					
Neuropsychiatric Inventory Questionnaire (NPI-Q) (66)	5	X					
Insomnia (Hamilton Rating Scale for Depression) (67)	2	X					
**Gait**							
Single-task gait speed		X					
Dual-task gait speed		X					
Dual-task gait cost		X					
Step length		X					
**Visual Symptoms**	10						
Diplopia		X					
Blink rate		X					
**Voice pitch**	5						
Pitch minimum		X					
Pitch maximum		X					
Pitch range		X					
Average pitch		X					
**Dysphagia**	1						
Sydney Swallowing Questionnaire (68)		X					
**Frailty**	5						
Edmonton Frail Scale (69)		X					
**Additional Measures**							
Saliva sample (APOE4 allele)	5	X					
**Medical History**	NA^*†*^						
Renal disease		X					
Previous stroke/TIA		X					
Atrial fibrillation		X					
Coronary artery disease		X					
Hypertension		X					
Peripheral vascular disease		X					
**Risk Scores**	NA^*†*^						
EuroSCORE II		X					
Society of Thoracic Surgery		X					
**Procedural Factors**	NA^*†*^						
Valve access type			X				
Procedure time			X				
**Population Characteristics**	NA						
Age		X					
Gender		X					
Height		X					
Weight		X					
Body mass index		X					
**Population Characteristics (collected at baseline)**	5						
Waist circumference		X					
Years in education		X					
Social engagement		X					
Physical activity		X					
**Approximate session durations (minutes)**		120	20	20	45	45	45

*CANTAB, Cambridge Neuropsychological Test Automated Battery; D1, day 1 post-TAVI; D2, day 2 post-TAVI; 3 M, 3-months post-TAVI; 6 M, 6-months post-TAVI; 12 M, 12-months post-TAVI; NA, Not applicable; TIA, transient ischemic attack*.

* *This assessment has parallel versions (or randomized aspects) to be used in follow-up sessions*.

† *Assessments are conducted/collected by the Royal Adelaide Hospital (administration, nursing staff and doctors) in the lead up to TAVI and during hospital stay, all other assessments to be conducted by researcher*.

##### Depression

The Geriatric Depression Scale (GDS), developed to detect level of depression in older populations (65), will be used to assess depression. The GDS consists of 15 items with response options of yes or no (i.e., “Are you in good spirits most of the time?”). Possible scores range from 0 to 15, with higher scores indicating greater likelihood of depression. Compared with diagnostic criteria, the GDS has high sensitivity (92%) and specificity (89%) (65).

##### Neuropsychiatric Symptoms

Neuropsychiatric symptoms will be evaluated using the Neuropsychiatric Inventory Questionnaire (NPI-Q) (66). This questionnaire is designed to be completed by the caregiver of a person with dementia. The caregiver is asked to note whether each of 12 neuropsychiatric symptoms (e.g., apathy/indifference, appetite/eating disturbances) have been present in the patient within the past month. If a symptom has been present, they then rate both the severity of the symptom (from 1 to 3, with higher scores indicating greater severity) and the distress it causes them (from 0 to 5, with higher scores indicating greater distress) (66). In this study, a family member or spouse of the participant will complete the NPI-Q. The person completing the NPI-Q must be without a clinical diagnosis of dementia, have sufficient English language and normal or corrected-to-normal vision (to ensure accuracy of report).

##### Insomnia

Insomnia will be assessed using the early, middle, and late insomnia items of the Hamilton Rating Scale for Depression (67). Each of these items is rated on a scale which ranges from 0 (absent) to 2 (severe). These early, middle and late insomnia items have been found to correlate highly (Spearman rho = 0.63, 0.49, and 0.49, respectively) with weekly averages for respective items measured using a sleep diary (70).

##### Gait

The assessment of gait will include single-task gait speed, dual-task gait speed, dual-task gait cost and step length. Each task will involve walking 6 meters, with participants able to use a walking aid if needed. Single-task gait speed is calculated as the distance (6 meters) divided by the time taken to walk the distance (in seconds). Dual-task gait speed is calculated in the same way as single-task gait speed, except participants count backwards from 100 (i.e., “100, 99, 98…”) while performing the walking task. Dual-task gait cost is calculated in the following way:

$$\begin{array}{l} \text{Dual task gait cost}=\frac{\text{Single task gait speed}-\text{Dual task gait speed}}{\text{Single task gait speed}}\\ \;\times 100 \end{array}$$

Step length (in cm) is calculated as the distance (600 cm) divided by the number of steps taken during the single-task gait assessment.

##### Diplopia

A protocol to assess presence of diplopia using an eye cover test was developed in discussion with a neuro-ophthalmologist. Participants' best corrected visual acuity (BCVA) will be assessed using a Snellen eye chart placed 6 meters away. A fixation letter which is 2 lines above the BCVA of a participant's worst eye will be chosen, and participants will be instructed to fixate on this throughout the eye cover test. The eye cover test will begin with the right eye being covered and then uncovered several times (holding for 1 s in each position), while watching the left eye for movement. The same process is then undertaken on the opposite eye. An alternating cover test is then performed, moving the occlude between the right and left eye (holding for 1 s over each) and watching for movement in either eye. Presence of double vision or blurry vision (by self-report) in addition to any eye movements (in right or left eye) during the eye cover task will be counted as presence of diplopia.

##### Blink Rate

The protocol for assessing eye blink rate was modeled based on the conversation task of Fitzpatrick et al. (49). Participants will be video recorded during 2 min of free speech, with prompts from the researcher only if needed. The average number of blinks per minute will be calculated from 3 separate trials of 20 s each from the 2-min recording. A blink is considered eye closure of at least 50%. Blink rate will be split into three equal groups for analyses, with middle being compared to low and high blink rate, in case of a non-linear relationship.

##### Voice

Measures of voice pitch (maximum, minimum, range and average) in Hz will be taken during sustained phonation /a/ at a comfortable pitch for the participant. Results will be based on an average taken from three separate recordings of 5 s duration each. All recordings used for average calculations will be free of background noise. The VoiceAnalyst app (iOS/Apple) will be used on an iPad (with microphone placed 20 cm away from participants' mouths) to take recordings and measure voice pitch. Male and female voice data will be analyzed separately.

##### Dysphagia (Difficulty Swallowing)

To assess difficulty swallowing, participants will be asked to respond to the question “Do you have any difficulty swallowing your saliva?” using a 10 cm visual analog scale, ranging from “No difficulty at all” (0 cm) to “Unable to swallow at all” (10 cm). This question was taken from the Sydney Swallowing Questionnaire (68).

##### Frailty

Frailty will be assessed using the Edmonton Frail Scale (69). This is a clinician-administered measure which comprises 10 domains. Some domains are assessed with performance-based items (e.g., the Clock Test for cognitive impairment), and others are quantified using questions with defined responses (e.g., “Do you use five or more prescription medications on a regular basis?” for medication use). Possible scores range from 0 to 17, with higher scores representing higher levels of frailty. The Edmonton Frail Scale has demonstrated good inter-rater reliability (κ = 0.77) and internal consistency (α = 0.62) (69). The Edmonton Frail Scale is also significantly correlated (*r* = 0.64) with the Geriatrician's Clinical Impression of Frailty (69).

##### APOE4 Allele

A saliva sample will be taken using an Oragene kit to test for presence or absence of the apolipoprotein E4 (APOE4) allele.

##### Medical History and Procedural Factors

Pertinent medical history, risk scores and procedural factors (see Table 2), based on those identified through previous research, will be collected from Royal Adelaide Hospital records.

#### Sample Characteristic Data

##### Population Characteristics

General population characteristics will also be collected in order to characterize the sample. These measures include age, gender, years in education (including primary, secondary and any further formal education), height, waist circumference, weight, and body mass index. Level of social engagement and physical activity will be collected by self-reported rating of the frequency participants see a member of their family or friend and take part in physical activity. Response options for these self-reports are daily, weekly, fortnightly, monthly, less than monthly, and never.

### Procedure

**Figure 1:**
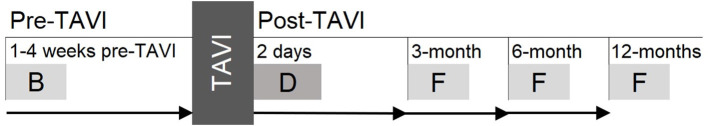
Study timeline showing pre-TAVI baseline as well as post-TAVI delirium assessment and three follow-ups. B, baseline; D, delirium assessment; F, follow-up.

The study timeline is outlined in Figure 1. Potential participants are approached in-hospital following the pre-procedural consult for their TAVI procedure. This typically occurs 1–4 weeks pre-TAVI. Informed consent is obtained from eligible and interested participants. Consenting participants then complete a baseline testing session (~2-h duration) in their home prior to their TAVI procedure. Multiple assessments (outlined in Table 2) are conducted at this baseline session. Prior to their procedure, a researcher also reviews the hospital medical history records for the participant to obtain relevant medical history data (see Table 2).

On the 2 days following their TAVI procedure, participants are assessed for delirium. This occurs either in-hospital or at their home, based on their location that day and if they have been discharged from hospital. A researcher also reviews the participant's medical record from the Royal Adelaide Hospital's records to obtain TAVI procedure information (see Table 2).

Participants are then contacted again at 3-, 6-, and 12-months post-TAVI to arrange follow-ups (~45-min duration). These sessions are once again conducted in participant's homes. If participants are unable to be contacted for follow-up, their next of kin is contacted.

### Data Analysis

To determine the relationship between the potential risk factors measured within this study and outcomes (primary and secondary), a series of univariate logistic and linear regressions will be conducted. Each predictor will be entered into a separate model for each primary outcome: presence vs. absence of delirium post-TAVI, as well as presence vs. absence or cognitive decline. The same will be done for secondary outcomes: cognition, mortality, quality of life, and activities of daily living at each follow-up (3-, 6-, and 12-months post-TAVI). Logistic regressions will be run for categorical outcomes (cognitive decline, delirium, and mortality) and linear regressions will be run for continuous outcomes (cognition, quality of life, activities of daily living). As this is an exploratory, effect-finding study, we will not correct for multiple comparisons and multivariable models will not be run. The purpose of this study is to propose variables to be taken forward into independent and larger samples, to develop TAVI-specific risk prediction tools for delirium and cognitive decline.

## Discussion

Risk prediction tools which accurately identify those at increased risk for post-procedural cognitive decline and delirium are enormously valuable. Early identification allows for timely diagnosis of these negative outcomes, which is crucial for their effective management. Additionally, pre-procedural risk identification is essential for the successful implementation of preventative programs or other multicomponent intervention (17). A meta-analysis has shown that the use of such multicomponent interventions, such as the hospital elder life program (71), can lead to a significant reduction in delirium incidence (OR = 0.47) (72). In order to develop such accurate and useful risk prediction tools for post-TAVI cognitive decline and delirium, we first must understand what factors to include. We are going to take a broad, inclusive and clinically relevant approach to take a step in this direction. However, our conclusions must be balanced against the limitations of our sample which has a bias toward individuals living in metropolitan South Australia (due to follow-up procedure logistics) and with a lower socio-economic status, only recruited from one public hospital. This is an effect-finding study using single-predictor regression models. As suggested by Lindroth et al. (73), future studies using multiple variables to develop a prediction model should consider utilizing lasso regression (or a similar penalized regression approach) in order to avoid overfitting. There has been limited and conflicting research investigating the utility of frailty measures in the prediction of delirium following TAVI (41–43), and even less research for cognitive decline. Measures of frailty are broad and vary greatly in the factors they encompass. We propose that by investigating individual factors drawn from frailty and aging literature, we can identify those with the best predictive utility. Future research can then implement these measures into new risk prediction models for TAVI, which can then be tested in larger samples.

## Ethics Statement

The studies involving human participants were reviewed and approved by the Central Adelaide Local Health Network (CALHN) Human Research Ethics Committee (Reference number: R20170916) and the University of South Australia Human Ethics Committee (Reference number: 200830). The patients/participants provided their written informed consent to participate in this study.

## Author Contributions

HK, DD, PP, and TL were involved in conception of the work. EG drafted the manuscript. All authors revised the work critically for important intellectual content and have read and approved the manuscript.

## Conflict of Interest

The authors declare that the research was conducted in the absence of any commercial or financial relationships that could be construed as a potential conflict of interest.

## Publisher's Note

All claims expressed in this article are solely those of the authors and do not necessarily represent those of their affiliated organizations, or those of the publisher, the editors and the reviewers. Any product that may be evaluated in this article, or claim that may be made by its manufacturer, is not guaranteed or endorsed by the publisher.

## Abbreviations

ACE-III: Addenbrooke's Cognitive Examination III
APOE4: Apolipoprotein E4
BCVA: Best Corrected Visual Acuity
CAM: Confusion Assessment Method
CANTAB: Cambridge Neuropsychological Test Automated Battery
EuroSCORE: European System for Cardiac Operative Risk Evaluation
GDS: Geriatric Depression Scale
ICU: Intensive Care Unit
MCI: Mild Cognitive Impairment
MDAS: Memorial Delirium Assessment Scale
MOT: Motor Orientation Task
NPI-Q: Neuropsychiatric Inventory Questionnaire
PRM: Pattern Recognition Memory
RTI: Reaction Time
STS: Society of Thoracic Surgery
TAVI: Transcatheter Aortic Valve Implantation.
